# Establishing a community of practice of researchers, practitioners, policy-makers and communities to sustainably manage environmental health risks in Ecuador

**DOI:** 10.1186/1472-698X-11-S2-S5

**Published:** 2011-11-08

**Authors:** Jerry M Spiegel, Jaime Breilh, Efrain Beltran, Jorge Parra, Fernanda Solis, Annalee Yassi, Alejandro Rojas, Elena Orrego, Bonnie Henry, William R Bowie, Laurie Pearce, Juan Gaibor, Patricio Velasquez, Miriam Concepcion, Margot Parkes

**Affiliations:** 1 University of British Columbia (UBC) – Global Health Research Program, School of Population and Public Health, Vancouver, British Columbia, Canada; 2 Universidad Andina Simón Bolívar, Quito, Ecuador; 3 Observatorio en Salud Colectiva, Ambiente y Sociedad, Organismo Andino de Salud, Quito, Ecuador; 4 Servicio nacional de control de los enfermedades transmitida por los artrópodos (SNEM) – Ministerio de Salud Pública, Machala, Ecuador; 5 Universidad Técnica de Machala, Machala, Ecuador; 6 Universidad de Cuenca, Cuenca, Ecuador; 7 UBC- Faculty of Land & Food Systems, Canada; 8 BC Centre for Disease Control, Vancouver, British Columbia, Canada; 9 UBC – Faculty of Medicine, Canada; 10 UBC - School of Community and Regional Planning, Canada; 11 Universidad Estatal de Bolívar, Guaranda, Ecuador; 12 Instituto Nacional de Higiene, Epidemiología y Microbiología, Havana, Cuba; 13 University of Northern British Columbia, Canada

## Abstract

**Background:**

The *Sustainably Managing Environmental Health Risk in Ecuador* project was launched in 2004 as a partnership linking a large Canadian university with leading Cuban and Mexican institutes to strengthen the capacities of four Ecuadorian universities for leading community-based learning and research in areas as diverse as pesticide poisoning, dengue control, water and sanitation, and disaster preparedness.

**Methods:**

In implementing curriculum and complementary innovations through application of an ecosystem approach to health, our interdisciplinary international team focused on the question: “Can strengthening of institutional capacities to support a community of practice of researchers, practitioners, policy-makers and communities produce positive health outcomes and improved capacities to sustainably translate knowledge?” To assess progress in achieving desired outcomes, we review results associated with the logic framework analysis used to guide the project, focusing on how a community of practice network has strengthened implementation, including follow-up tracking of program trainees and presentation of two specific case studies.

**Results:**

By 2009, train-the-trainer project initiation involved 27 participatory action research Master’s theses in 15 communities where 1200 community learners participated in the implementation of associated interventions. This led to establishment of innovative Ecuadorian-led master’s and doctoral programs, and a Population Health Observatory on Collective Health, Environment and Society for the Andean region based at the Universidad Andina Simon Bolivar. Building on this network, numerous initiatives were begun, such as an internationally funded research project to strengthen dengue control in the coastal community of Machala, and establishment of a local community eco-health centre focusing on determinants of health near Cuenca.

**Discussion:**

Strengthening capabilities for producing and applying knowledge through direct engagement with affected populations and decision-makers provides a fertile basis for consolidating capacities to act on a larger scale. This can facilitate the capturing of benefits from the “top down” (in consolidating institutional commitments) and the “bottom up” (to achieve local results).

**Conclusions:**

Alliances of academic and non-academic partners from the South and North provide a promising orientation for learning together about ways of addressing negative trends of development. Assessing the impacts and sustainability of such processes, however, requires longer term monitoring of results and related challenges.

## Background

In 2004, the *Sustainably Managing Environmental Health Risks in Ecuador* (*Ecuador Eco-Health*) project was launched in response to concerns that deteriorating ecological and social conditions in this low-income South American country were aggravating the threats to health being experienced by vulnerable populations during a period of intensifying global integration [1]. Amid the widespread poverty and disparities present in Ecuador, attention focused on the systemic circumstances that could be affecting health, such as the contamination of land, air and water by intensive application of agro-toxins [2] or poorly controlled mining activities [3], as well as by deteriorating living conditions and inadequate water and sanitation in both urban and rural settings [4]. With funding from the Canadian International Development Agency (CIDA)’s University Partnerships in Cooperation and Development program, an ambitious multi-faceted project was launched to strengthen capacities for designing, conducting and evaluating interventions to contribute to sustainable solutions. The focus was on developing knowledge, skills and attitudes for conducting rigorously designed action research projects, building on the potential of international collaborations to support this [5-7].

The project originated from 4 distinct global trends that intensified in the 1980s and 1990s:

i) Increasing **disparities and negative consequences of development** resulting from economic globalization [8];

ii) **Recognition of the complexity of factors in social and ecological systems** that contribute to human health outcomes [9];

iii) **Introspection into the role of the university**, especially the ability to effectively address community concerns [10]; and

iv) Insights into the **limits of traditional forms of pedagogy** and the power of interactive learning [11].

Chronic underfunding of public universities in the atmosphere of fiscal restraint that predominated in this era was a further global pressure of consequence [12] as these institutions concurrently grappled with new demands to address intensifying social and environmental concerns amid great unevenness in the capabilities to do so. In Ecuador, this was complicated by the challenge of interculturality (an estimated 25-30% of the population identify as indigenous) as well as existing low investment in the health and education sectors. To address such challenges, models of collaboration between universities in low and high income countries that emphasize local leadership and ownership are receiving growing attention [13,14], especially as an alternative to training approaches that have served to encourage migration away from low and middle income country settings.

## The research

While our initiative itself focused on more systematic pursuit of action research investigations that incorporated *transdisciplinarity*, *participation*, *equity* and *sustainability* (i.e. the framework and methodologies associated with an “ecosystem approach to human health” promoted by Canada’s International Development Research Centre [IDRC]) [15], the creation of a national network with international links was, in and of itself, conceived to be an intervention. A central research question was thus embedded in the undertaking: “Can strengthening institutional capacities to support a community of practice of researchers, practitioners, policymakers and communities produce sustainable health outcomes and capacities to effectively translate knowledge?” This in fact reflected an orientation supporting the creation of such international networks to encourage the application of eco-health approaches [16]. We sought to explicitly concentrate on a national scale, but with the support of international partners.

We proceeded with the understanding that Communities of Practice (CoP) are groups of people who share a concern, a set of problems, or a passion about a topic, and who deepen their knowledge and expertise in this area by interacting on an ongoing basis [17]. A systematic review on the use of CoPs in business and healthcare observed that while specific structures of groups varied greatly, they share four characteristics: social interaction among members, knowledge sharing, knowledge creation, and identity building [18]. This orientation to capacity-building was itself embraced as integral to the academic programs at the core of the “Ecuador Eco-Health” initiative.

A comprehensive Logic Framework Analysis was developed to guide activities, specifying outputs (such as established programs and their curricula, numbers of graduates etc.), outcomes (institutional strengthening indicators such as accredited programs, qualified faculty, obtained funding for new collaborative research projects) and impacts (improvements in human and ecological health achieved, with particular attention to health equity). Recognizing the innovative character of the intervention, explicit focus was applied to observe whether synergies could be established to reinforce processes and structures capable of effectively and sustainably achieving desired outcomes and impacts.

What was at stake, in this regard, over the initial six years of the initiative (2004-2010) has principally been a testing of the feasibility of consolidating institutional entities capable of knowledge translation to improve health equity. To monitor progress, documentation of processes, activities and participants (including community and policy-makers) has been consistently maintained, including follow-ups of graduates and the action research projects (by co-author EO) at 6 month intervals. In addition to providing a summary of this experience, we present two case studies to more vividly illustrate the interactive benefits enabled by a community of practice, along with a discussion of challenges that have been and continue to be part of this process.

## Results and outcomes

The first phase (2004-2008) of our *Ecuador Eco-Health* initiative focused on implementing a “train the trainer” perspective through a nationally accredited “Master’s in Health with an Eco-system Focus”. Explicit criteria were applied at different levels to support this orientation:

i) Selection of participating institutions was designed to include a university with well-established academic traditions (U of Cuenca, founded in 1867) and 2 relatively new universities that lacked strong research capacities, but where serious problems persisted: intensive resource extraction and poverty (Technical U of Machala, founded in 1969); indigenous rural populations and poverty (U of Bolivar, founded in 1977).

ii) Selection of students was based on criteria that explicitly weighed academic disciplinary aptitudes alongside capacity for undertaking community-based interventions and knowledge translation, as well as cultural diversity, producing a first group of 30 students who were interdisciplinary, interregional, and intercultural with 5 indigenous community leaders. Additionally, 5 of the 10 selected by each university had to have university faculty positions and the promise of continued faculty positions after completion of their program. The expectation was that these latter 15 individuals would play a key role in maintaining appropriate Eco-health programs at each of the universities.

iii) Criteria for thesis research were explicitly adopted to include not only 1) rigorous and clear methodology, but to insist that there be 2) clear research and impact objectives; as well as 3) focus on relationships between human health, ecosystem health, and community wellbeing; 4) significant community participation; and 5) collaborative relationships with other students, key stakeholders, and the community.

An innovative curriculum to support interactive community-based learning for the first cohort of the Master’s Program was developed and implemented by the international team led by co-authors JS and AY and established Ecuadorian researchers in the area led by co-author JB, with the coordination of co-author MP. To reinforce the role played by local university thesis directors, who were only becoming acquainted with a new action research orientation amid their other obligations, a dedicated team to support thesis preparation was established and coordinated by co-author EO. As a result, 27 participatory action research Master’s theses were successfully completed in 15 communities by 2009, involving over 1200 community learners. Through this collective experience, reflections on inadequacies of current theoretical and methodological approaches fuelled a focus on the social *determination* of health, observable in distinct contexts, and not the excessive examination of isolated determinants.

*From a “top-down” perspective*, this experience served as a crucible for establishing new training programs led by Ecuadorian universities with international reinforcement. A second Master’s program cohort was initiated by the University of Cuenca (led by co-author JP) in 2009. When the Ecuadorian academic director for the inaugural Master’s program (co-author JB) was appointed director of the health area of University Andina Simon Bolivar (UASB) in 2008, the consortium expanded to support the launch the following year of an innovative Doctoral program on “Collective Health, Environment and Society” to provide a strong foundation for the emerging national network. The Universities of Bolivar (co-author JG, a professor who is a Master’s graduate and current UASB PhD student) and Machala (co-author PV, a professor who is a UBC PhD graduate with support of the Ecuador Eco-Health program) meanwhile are preparing certificate courses to be operated in 2011 to build capacity in priority areas for their context, and workshops with active practitioners are being conducted in areas such as disaster preparedness in collaboration with co-author LP.

Also in 2009, an innovative way to link directly with policy-makers was begun through a direct alliance between the UASB PhD program and the *Organismo Andino de Salud*, now led by the former Ecuadorian Minister of Health, which represents Andean Region governments affiliated with the Pan American Health Organization. The “Observatorio Regional de Salud Colectiva, Ambiente y Sociedad” [Regional Observatory on Collective Health, Environment and Society] (led by co-author JB with participation of members of the international team as a part of the group of professors delivering the PhD program modules including co-authors JS, AY, AR and other international experts) identifies priority research areas capable of macro and meta level analyses of evidence related to health concerns and policies at a national and sub-continent / regional level [19]. These are directly linked with the PhD initiative that itself has an Andean (i.e. multi-country) charter – providing a strong policy-relevant orientation for the 18 action-research doctoral investigations that are now underway.

*From a “bottom-up” perspective*, locally established action research projects have laid foundations for larger scale applications engaging broader arrays of policy makers and funding agencies to enable further knowledge translation and innovation. Some examples of local initiatives that led to sustained impact include the following: In the urban community of Machala, three feasibility projects were undertaken as Master’s theses to strengthen community based dengue prevention and control, through direct involvement of school children, health promoters and community members as well as the pursuit of alternatives to heavy pesticides use. Building on this foundation (established with assistance from co-authors WB, BH and AY), a 3 year trial applying a comprehensive integrated surveillance program was launched in 2010 with funding from the WHO Tropical Disease Research Program (TDR) and IDRC, notably with the 3 Principal Investigators being i) a Master’s program graduate who heads the province’s Vector Disease Control program (co-author EB); ii) the PhD program director (co-author JB); and iii) the UBC-based *Ecuador Eco-Health* project director (lead author JS), with active involvement of Canadian and Ecuadorian graduate students. The provincial Ministry of Health is directly involved and committed to province-wide scaling up once results are evaluated with an active participation of local school boards, health units and community organizations.

In the rural setting of San Jose de Balzay, a community heavily involved in the artisanal production of clay tiles and roofing materials, lead poisoning (from glaze used in the production process) was diagnosed and alternative materials and work practices incorporated to alleviate this problem as the result of a student thesis in the first master’s cohort. By working with local community organizations and an active national Non-Governmental Organization, Acción Ecológica, the University of Cuenca, in partnership with the UASB and UBC launched (in December 2010) a new form of “eco-health centre” (*Clinica Ambiental del Sur*) based on the linking of prevention at a population level with primary care clinical attention in underserviced areas. It is noteworthy that as new initiatives are being undertaken, opportunities are being increasingly created for women researchers who have been previously under-represented in the ranks of the leadership. For example, direction of the *Clinica* initiative as well as the second cohort of the Masters program was assumed by a young female PhD trainee (co-author FS) in 2010.

A further action research program addressing food security, food sovereignty and health equity has been initiated, with submissions for international peer-reviewed funding in early 2011, by co-authors JS, AR and JB, along with other members of the authorship team together with additional community and policy-maker partners–with funding for 5 years approved to begin in late 2011. To reinforce concentration on practical applications of knowledge, this group includes the national Ministry of Health, the Ministry of Agriculture (reinforced by the hiring of a Master’s program graduate as national director of the irrigation program), the Ecuadorian section of the development bank Banco del Sur which is interested in providing credit for more sustainable and equitable forms of agriculture, the national organization of small-scale indigenous and non-indigenous farmers (FENOCIN), and peasant coastal producers involved in domestic and export-oriented production (UROCAL).

Innovative methods to teach and learn constitute an integral part of the Ecuador Eco-Health initiative through the team’s explicit pursuit of a strategy emphasizing participation through the formation of a community of learners combined with problem-based learning centered on contributing to beneficial community impacts. Knowledge sharing has fundamentally taken place within the dynamics of difference (three or more cultures, half a dozen disciplines, distinct paradigms, three languages, and the great diversity of the Ecuadorian regions). This approach was vividly evident in repeated showings on national television of a video on the benefits of pursuing a more integrated intercultural approach to childbirth in indigenous communities based on the Master’s thesis of an indigenous nurse with the national Department of Indigenous Health. This initiative seeks to incorporate both traditional and modern practices to improve maternal health. With UBC partners, this team is actively pursuing international funding for a network of communities and government agencies that will make timely technical assistance possible through innovative use of communication technologies alongside improved local mobile infrastructure.

The results to date from the Ecuador Eco-health project indicate that action research oriented to health equity is quite feasible if there is a commitment from both the “top down” and the “bottom up”, engaging local policy makers and practitioners in the research process, and then broadening the partnerships to consider scaling up. The effectiveness of these efforts in terms of health outcome will continue to be studied in the coming years.

## The partnership

From the very beginning, collaboration was conceptualized from a “North-South-South” perspective. In fact, the feasibility of what would eventually become the Ecuador Eco-Health project was initially suggested in 2001 by Ecuadorian participants at a Canadian-Cuban Eco-Health workshop in Havana, Cuba, including co-author MC, providing the foundation for subsequently shaping how opportunities evolved. To support sustainability of the networks being created, two leading Latin American institutions expert in environmental health, INHEM in Cuba and INSP in Mexico, have been members of the partnership from the earliest days.

The concept of building on pre-existing capabilities was central from the beginning as a key characteristic of the national network that would emerge in Ecuador itself, linking centres with different strengths and experiences. External Canadian involvement *de facto* served as a catalyst for initiating new approaches for pursuing interdisciplinary research and multi-university partnerships, with more direct engagement of communities and policy-makers. This was especially evident when the Ecuadorian university partners acknowledged that adequate experience was initially lacking in their ranks to provide faculty direction and support. When one university involved in the original planning backed out in 2004 because of a failure to consolidate commitment to the emerging direction, an Ecuadorian NGO with extensive experience and expertise in conducting eco-health action research (led by co-author JB) was invited to join the consortium to provide national academic direction that built on similar traditions whose roots were distinctly Latin American and internationally recognized [20]. In this emerging partnership, the involvement of a wide range of disciplines at a university of the scale of UBC facilitated comprehensive pursuit of a transdisciplinary approach in the Ecuadorian context. To help overcome the challenges of language and culture, the Canadian team was furthermore able to take advantage of Vancouver’s multi-cultural character itself to directly engage Canadians of Ecuadorian and other Latin American ancestry in the team!

To provide a unifying force amid considerable diversity, strong allegiance to the project’s vision has been likened by team members to the expression of “*amor a la camiseta*” – love of the jersey (for one’s hometown soccer team) that can overcome competing interests. As suggested by the importance accorded to “identity building” in communities of practice [18], such commitment must be critically considered if there is indeed to be an institutionalization of the values being established.

## Challenges and successes

Pursuit of the Ecuador Eco-Health initiative was a very ambitious undertaking – requiring moving from unidisciplinary to transdisciplinary teams, combining intercultural perspectives and moving from research for inquiry sake to impact-oriented investigation, while maintaining rigor in methods. This was, of course, in addition to challenges inherent in all north-south collaborations. As no existing requirements were in place to govern research ethics in the Ecuadorian context, internal processes were put in place for research undertaken. Notably, in pursuing this course, a number of students were inspired by the experience of Aboriginal communities in Canada to ensure respectful relationships [22], and formally included obligations to share research findings with communities as an explicit part of public meetings that were held with the involved communities.

A paramount achievement realized over the course of program implementation involved transforming the model for doctoral training for the emerging network from a traditional model involving studying abroad to one that could be undertaken directly at an Ecuadorian university. This was facilitated through the 2008 signing of a Memorandum of Understanding between UBC and UASB which ensured the involvement of international professors so that accreditation requirements could be satisfactorily met. Ecuadorian PhD students have thus been permitted to continue to work in their home settings while reinforcing and being reinforced by the emerging community of practice that is being established through the institutional strengthening efforts described above. By providing this more sustainable alternative, the challenge of reintegration following years of absence can be avoided; something that our program’s one foreign trained PhD graduate (co-author PV) is now in the process of addressing. This national PhD design, furthermore, has enabled one of the five indigenous members of the first cohort to continue to pursue a doctorate focused on food security challenges in his community.

While most graduates are managing to apply their skills to their pre-existing work settings or moving to new opportunities, we have observed that a number would definitely have benefitted from their employers having a greater appreciation and support of the orientation that the program was presenting. In this sense, a community of practice network provides a more comprehensive way for reinforcing implementation and adaptation of the changes in application of knowledge that are envisioned. An encouraging recognition of the eco-health approach has been exhibited by the Faculty of Medicine at the University of Cuenca who have committed to further training of their faculty in participatory research and teaching methods as well as to broader applications of an eco-system approach to health.

The successes achieved through international collaborations nevertheless remain, above all, dependent on the engagement of rooted national expertise, as well as the embracing of different types of knowledge (e.g. local, ancestral) in a coherent conceptual framework. The holistic indigenous vision of health (called *sumak kawsay* in the Kichwa language – or “good living” in English) that is framed as the overriding social purpose in Ecuador’s Constitution of 2008 [21] is providing an especially timely opportunity for our project to contribute to this mission. As the global quest of creating more sustainable alternatives is itself calling into question the values underpinning Western development models, our team is especially enthusiastic in pursuing opportunities to explore how alternatives can be created.

Appreciating different cosmologies and their links to ways of seeing and knowing has, in addition, made our team especially sensitive to the challenge of working with graduate students whose knowledge system communicate more strongly orally and metaphorically than conceptually and abstractly. This has prompted the question: Should they be allowed to deliver integrative products other than traditional academic theses? And if so, what standards of excellence should be used - only those conventionally accepted in Western academia or a plurality reflecting interculturality? Recognition of project-based experiences as an acceptable basis for master’s level education, an approach being increasingly accepted in Canadian universities, has been actively discussed – but, for the time being, has been rejected for application in the Ecuadorian context. However, this will be the subject of further review in assessing ways to better support intercultural learning.

In many ways it is premature to pass judgement on the question guiding this undertaking, namely the effectiveness and sustainability of applying a community of practice orientation to strengthening capacities to conduct intervention-oriented research. Nevertheless, the Ecuador Eco-Health initiative has vividly demonstrated how alliances of academic and non academic partners from the South and North provide a promising ways to learn together about ways of addressing negatives trends of development. Assessing the legacy of such processes, however, requires longer term monitoring of results and related challenges.

While it is difficult to measure the long-term impact on health of the communities involved in the research, the number of trained individuals now continuing to implement internationally-funded peer-reviewed research is itself a testimony to success of this effort thus far. Accordingly, we strongly feel that the consolidation of a community of practice beyond the end of the formal funded capacity development period augurs well for sustained application of the core values and vision that was the original goal of our endeavour.

## List of abbreviations used

CIDA: Canadian International Development Agency; FENOCIN: National Confederation of Rural, Indigenous and Afro- Ecuadorian Organizations; IDRC: Canada’s International Development Research Centre; INHEM: Instituto Nacional de Higiene, Epidemiologia y Microbiología; INSP: Instituto Nacional de Salud Pública; PI: Primary Investigator; TDR: Tropical Disease Research; WHO: World Health Organization; UASB: University Andina Simon Bolívar; UROCAL: Coastal Regional Union of Peasant Organizations; UBC: University of British Columbia.

## Competing interests

All authors declare that they have no competing interests.

## Authors' contributions

JS and AY conceived the initial project, and developed it from the earliest phase with co-authors LP and WB, and shortly thereafter with the close collaboration of coauthor JB who spearheaded the development and implementation of the emerging community of practice in Ecuador, including the shaping of academic research programs to support this. Co-authors AR, MP, BH and MC have been actively involved as members of the international team contributing to the curriculum development, teaching and research activities involved in the project. Co-author EO led the team supporting the pursuit and writing up of thesis research. Co-authors EB, JP, FS, PV and JG have actively involved as both trainees and leaders of research, teaching and research application activities in Ecuador. JS prepared the first draft of this manuscript in collaboration with co-author JB. All authors helped to write and revise this manuscript.
